# Race- and gender-based under-representation of creative contributors: art, fashion, film, and music

**DOI:** 10.1057/s41599-022-01239-9

**Published:** 2022-06-29

**Authors:** Chad M. Topaz, Jude Higdon, Avriel Epps-Darling, Ethan Siau, Harper Kerkhoff, Shivani Mendiratta, Eric Young

**Affiliations:** 1 Institute for the Quantitative Study of Inclusion, Diversity, and Equity, Williamstown, MA USA; 2 Williams College, Williamstown, MA USA; 3 Bennington College, Bennington, VT USA; 4 Harvard University, Cambridge, MA USA; 5 Northwestern University, Evanston, IL USA; 6 Brown University, Providence, RI USA; 7 University of Colorado, Boulder, CO USA

**Keywords:** Cultural and media studies, Cultural and media studies, Sociology

## Abstract

Motivated by the well-established benefits to society of artistic creation and of demographic diversity, we investigate the gender and racial/ethnic composition of influential contributors to four creative domains. Women make up 51% of the U.S. population but are underrepresented at influential levels of contemporary art (28%), high fashion (45%), box office film (27%), and popular music (17%). Marginalized racial/ethnic groups make up 39% of the U.S. population yet comprise approximately half that figure in contemporary art (22%), high fashion (22%), and box office film (19%). Black musical artists have higher representation (48%), though higher representation does not equate with equity and inclusion. As for intersecting identities, white men are overrepresented in all four domains by factors ranging from 1.4 to 2 as compared to the U.S. population, and most other gender-racial/ethnic groups are further minoritized. Our study is the first comprehensive, comparative, empirical look at intersecting identities across creative fields. The exclusion of marginalized individuals, including those who are women, American Indian/Alaska Native, Asian, Black, Latinx, and Native Hawaiian/Pacific Islander, is severe. The lack of self-expressed demographic data is a challenge, as is the erasure of certain identity groups from the American Community Survey, including agender, gender noncomforming, nonbinary, and transgender individuals. These are challenges that, if addressed, would enhance our collective understanding of diversity in creative fields.

## Introduction

The creation of art is a universal human behavior that benefits individuals and, collectively, society (Dissanayake, 2015). Art can unite, educate, support health, enhance well-being, boost economies, and more (Arts Council England, 2014; Elsen, 1976; Kaimal et al., 2016; Siegesmund, 1998). For some, ethical imperatives speak to the importance of diversity in creative fields. This imperative is two-sided. It encompasses both access to careers for people from underrepresented or excluded groups and the ability of marginalized people to see creative output that mirrors their identities and experiences. Beyond any ethical imperatives, diversity matters because it enhances the aforementioned benefits of art (Arts Council England, 2019). That is to say, these benefits increase when there are no barriers to individuals wishing to create artistic output, and when audiences choose to engage with output that matters to them. Unfortunately, as empirical research has recently begun to highlight, reality falls short of this ideal.

Our current study strives to move the conversation about exclusion in the creative arts briskly forward. While there may be instinctual or anecdotal understanding of the severity of under-representation of minoritized groups within creative fields, empirical evidence is still scarce. Thus, we take a broad look at the socially inferred demographic identities of individuals across four creative domains in the present day: contemporary art, fashion, film, and popular music. Moreover, we focus on the levels of these fields that might traditionally be thought of as being influential: major U.S. art museums, high fashion runway shows, Hollywood box office films, and top popular music charts.

Our work makes four high-level contributions. First, by bringing together assessments from these fields, we are for the first time able to highlight similarities and differences among them. Second, we study gender and race/ethnicity jointly in each of these fields, enabling us to assess intersecting identities. Third, we make comparisons between the socially inferred demographics of creative contributors and the demographics of the U.S. population, emphasizing degrees of under-representation for certain groups. Finally, we highlight a transparent and semi-automated methodology so that our study could be repeated over time in order to assess change.

Ours is neither a psychological, sociological, nor organization-theoretical work meant to elucidate mechanisms of under-representation. Rather, the purpose of our observational study, which uses a convenience sample, is to identify and quantify loci of under-representation both in terms of specific creative fields and demographic groups. Our work has important limitations, and we caution the reader to pay close attention to these as discussed throughout and as summarized in section “Conclusion.”

Based on our study of 2229 contemporary artists, 889 fashion designers, 1580 film cast/crew, and 221 popular musicians, our primary results are as follows. While women make up 51% of the U.S. population, we find that they are underrepresented in contemporary art (28%), fashion (45%), box office film (27%), and popular music (17%). Minority racial/ethnic groups make up 39% of the U.S. population yet barely comprise half that in contemporary art (22%), fashion (22%), and film (19%). Black musical artists have higher representation (48%), though higher representation does not equate with equity and inclusion. As for intersecting identities, white men are overrepresented in all four domains by factors ranging from 1.4 to 2, and most other gender-racial/ethnic groups are further minoritized.

## Background

Our empirical study is part of a small but growing research landscape related to diversity and representation in arts and media. Below, we review selected scholarship in each of four creative domains, namely contemporary art, fashion, box office film, and popular music, focusing on the importance of diversity in these fields and on existing empirical results.

### Contemporary art

In 1986, a collective of anonymous artists known as the Guerrilla Girls released a report card tabulating the number of women artists displayed in two successive years at each of 17 commercial art galleries (Guerrilla Girls, 1986). These tallies revealed a severe under-representation of women, which the Guerilla Girls supplemented with written comments such as “still unsatisfactory,” and “boy crazy.” The report card served as a call to action for equity, diversity, and inclusion in the art world. Decades later, artists like Micol Hebron and the anonymous collective Pussy Galore have carried on in the tradition of the Guerrilla Girls. For instance, on a 2015 tally of 34 galleries, the median gallery had 29% women artists (Buffenstein, 2016).

The diversity landscape is also grim at auction. Artwork produced by women represented a meager 2% of auction sales between January 2008 and May 2019 according to one study that examined over 400 auctions (Halperin and Burns, 2019). The same study found that of those 2%, nearly half of the sales were for the works of just five artists: Yayoi Kusama, Joan Mitchell, Louise Bourgeois, Georgia O’Keefe, and Agnes Martin. This concentration of interest in such a small group suggests that opportunities for women artists are even more limited than previously imagined. A study of 1.5 million auction transactions in 45 countries found that relative to paintings by men, there is a 48% discount in prices for paintings made by women (Adams et al., 2018). The situation is similar for racial/ethnic diversity. For example, pieces by African American artists constituted only 2.3% of art acquisitions and gifts from 2008 through 2018. Furthermore, only 7.7% of commercial gallery exhibitions during that time period focused on work by African American creators (Halperin and Burns, 2019).

Over 30 years later, and inspired by the groundbreaking efforts of the Guerrilla Girls, one study quantified the minoritization of artists within the collections of art museums. This 2019 study examining 18 high-profile museums across the U.S. inferred 85% of artists to be white and 87% to be men (Topaz et al., 2019). While that study looked at overall catalog holdings, another study focused on special exhibitions. Of approximately 2000 shows surveyed, 10.9% were devoted to Black artists, 6.6% to Asian and Asian American artists, 5.5% to Latin American and Latinx artists, and 0.1% to Native American and indigenous artists. Only four shows featured artists using they/them pronouns at the time of exhibit (Greenberger, 2019).

### Fashion

Diversity in fashion, or lack thereof, has tangible effects. One of the first streams of diversity-related research in fashion focused on body type. Exposure to thin-ideal bodies has negative psychological impacts for women, as does exposure to muscular, hypermasculinized bodies for men. Conversely, exposure to fashion models of a diversity of body sizes and shapes can promote positive body image in viewers (Barry, 2014; Borland and Akram, 2007; Diedrichs and Lee, 2010; Grabe et al., 2008). More recently, popular attention has turned to other axes of diversity in fashion, with Vogue publishing a timeline of breakthroughs in diversity during the 2010s (Okwodu, 2019).

Despite some breakthroughs, the fashion industry remains fairly homogeneous. One study of fashion design and product development textbooks found that of 3124 women models pictured, merely 15% were people of color (Reddy-Best et al., 2018). Another study examined race/ethnicity of models for the first ten looks presented by Calvin Klein, Chanel, and Versace during New York fashion week from 1992 through 2019. A full 88% were white (McDowell, 2019). More broadly, The Fashion Spot publishes “Diversity Reports” every few months, dating back to June, 2014. These reports study the New York, Paris, and London fashion weeks to assess diversity of models on various axes, including race/ethnicity. The representation of white models remains high, at 60% for spring 2021, though it is down from 85% in 2015 (Schimminger, 2020).

While the diversity of models is now a subject of regular study, less is known about other participants in the fashion industry. At the Paris, New York, London, and Milan fashion weeks during spring 2020, the percentages of white designers hosting the shows were 58%, 66%, 70%, and 88%, respectively (Kent, 2019). To get an overall sense of the field, the Council of Fashion Designers of America surveyed 50 executives at 30 major fashion companies, asking their opinions on diversity within the industry. These executives rated the industry as “average” (Council of Fashion Designers of America and PVH Corp., 2019). Finally, in one study, the researcher worked as a model while conducting a two and a half year long ethnography. She concluded that decision-makers in fashion “face intense market uncertainty,” and as a strategic response, “they rely on conventions, imitation, and stereotypes to guide their decisions” (Mears, 2010).

### Film

Gender and racial/ethnic identities in film play a key role in shaping our society, and have the potential to amplify sexism and racism (Eschholz et al., 2002). Diversity among film actors, directors, producers, writers, and other contributors has been lacking since the start of the film industry (Amaral et al., 2020). Moreover, since its inception, the film industry has been used to justify racial hierarchies and bolster white supremacy (Erigha, 2019). At the same time, recent evidence suggests some public desire for films to showcase more diversity (King et al., 2021).

Proximate to Hollywood, universities in southern California have been leaders in studying diversity in the film industry (Hunt and Ramón, 2020; Smith et al., 2020). For example, a 2020 study of the University of Southern California’s Annenberg Inclusion Initiative examines the top 100 grossing films from 2006–2019 (Smith et al., 2020). For films from 2019, merely 34% of speaking characters were women, a share that does not differ substantially from the 30% seen in 2007. At the same time, for the data from 2019, 66% of speaking characters were white. Of the 100 films that year, 15 had no speaking characters who were Black, 44 had none who were Hispanic/Latinx, and 36 had none who were Asian. There was almost complete erasure of characters with native, Middle Eastern/North African, and other minoritized racial/ethnic backgrounds.

Homogeneity of gender and race/ethnicity is not merely found in front of the camera. Across the 1,300 films studied in (Smith et al., 2020), the gender ratio for directors was 12.2 men for every one woman. The same study finds that films directed by women feature women as leading characters at more than double the rate seen in films directed by men. Combined, these two results hint at a vicious cycle of exclusion. As for race/ethnicity, more than 80% of the directors of major films from 2019 were identified as white. However as with gender, the behind-the-camera demographics influence those in front of the camera. While only 12.1% of speaking characters were Black in films directed by non-Black directors, the figure was 53% for films with Black directors.

### Music

As in other creative domains, the identities represented in popular music have real impacts. In one study, undergraduate students were exposed to Top 40 pop music (including Gwen Stefani, Justin Timberlake, Akon, and Rihanna), mainstream rock (including Foo Fighters and Radiohead), and self-identified white power rock (Prussian Blue, Max Resist). The subjects were then asked to distribute funding in a hypothetical situation for student groups on campus of various races. In the post-test experiment, white groups were favored more after exposure to mainstream and white power rock as compared to Top 40 music (LaMarre et al., 2012).

Inequality in popular music can start at the corporate level, where decision-makers have the power to control content that reaches consumers. A study of 4,500 record companies found that more than 85% showed a history of primarily signing men, while only 35% signed any women musicians at all (Wang and Horvát, 2019). In the United Kingdom’s music industry, women fill 60% of intern and entry level roles, but merely 30% of executive level roles (Njoke-Goodwin et al., 2020). Pay inequity is common, with major labels such as Sony, Warner, and Universal paying men 34% more than women on average across all levels of the businesses (Jones, 2018).

As they also do for the film industry, the Annenberg Inclusion Initiative compiles annual statistics on diversity in popular music (Smith et al., 2021). Their study surveys the gender and race/ethnicity of musicians and producers across 100 popular songs per year from 2012 through 2020. Across these years, the prevalence of women musicians never reached 30%, and this was merely 20% in 2019. Across the same time period, women accounted for merely 13% of Grammy nominees. As for people of color, representation amongst musicians with top songs increased from a low of 31% in 2013 to 59% in 2020. The Annenberg report does not comment on representation of people of color overall amongst Grammy winners, but does note that of the 182 women nominated in the past 9 years, merely 39% were from minoritized racial/ethnic groups.

## Methodology

We study over 4700 creative contributors within contemporary art, high fashion, box office film, and popular music in the United States. We begin in section “Crowdsourcing” with a description of Amazon Mechanical Turk, a tool that we use throughout our study. In section “Identity,” we describe the demographic characteristics we are interested in, namely gender and race/ethnicity. Our goal is not to determine individuals’ true identities along these axes (as this is not possible without the individuals’ self-stated identifications) but rather, society’s ascription of them, and we justify this approach in detail. As described in section “Scope of study,” the starting points for our study are contributors from four domains: contemporary artists whose works appear in certain high-profile museums; fashion designers whose clothing has been shown in major fashion shows; cast and crew of top-grossing box office films; and popular musicians who have appeared on top charts. The data for contemporary artists comes from a prior study (Topaz et al., 2019). For the other three creative domains, we gather new data on creative activity in 2018 and 2019. Owing to the COVID-19 pandemic, 2020 and 2021 may be anomalous years and we do not study them. Section “Crowdsourced demographic inference” provides more detail on social inference of demographics.

### Crowdsourcing

Crowdsourcing plays a key role in our data collection process. We use Amazon Mechanical Turk (MTurk). MTurk is a crowdsourcing labor platform where requesters post tasks that workers can complete for a monetary fee. These tasks are called *HITs*, which stands for Human Intelligence Tasks. HITs are tasks that can be completed by humans relatively easily, but that are difficult or impossible to program a computer to complete. After a worker completes a task, the requester can review the completed work and approve payment. The use of MTurk for studies of inferred demographic diversity has been established, for example, in (Topaz and Sen, 2016; Topaz et al., 2019, 2020).

We take several steps to promote data quality. First, we restrict access to our HITs so that they are only available to workers who have previously completed at least 1000 HITs on MTurk, and who have at least a 99% approval rate for that previous work. Second, because our study uses demographic characterizations that are common in the United States, and because it addresses creative contributors who are largely located within the United States, we restrict our HITs so that they are only available to workers located in the United States. Third, we have each individual in our study researched independently by at least four workers. Moreover, finally, for each axis of diversity, each worker provides a confidence rating for their response, and we use these ratings to weight responses in order to achieve a better description of the crowd’s consensus.

### Identity

We believe that the only true source of knowledge about an individual’s identities is the individual. To know creative contributors’ true identities, one would need to find public self-affirmations made by them, or request their demographics via a survey, which would likely have a low response rate.

Given the challenge of discovering true identities, we instead study society’s perception of these identities. We will use socially inferred demographic characteristics as rough proxies for identity, while bearing in mind that they cannot be assumed to be the same. This limitation speaks to the value that self-identified demographic information would have. Lacking such information, we proceed, albeit with caution, in order to move towards a quantitative understanding of under-representation in creative fields.

As an ancillary matter, our work may speak to *perceptive discrimination*, that is, discrimination based on the perception that an individual is a member of a particular identity group. For a review of perceptive discrimination, see (Maril and Gill, 2018), and for empirical studies demonstrating this effect for race and gender, see (Bertrand and Mullainathan, 2004; Moss-Racusin et al., 2012). In our study, we observe low participation of certain demographic groups, and this low participation is likely the result of both structural and interpersonal factors. For example, consider the race/ethnicity of actors in Hollywood films. A Black person may be more likely to face broad, structural challenges to becoming an actor, for example, intergenerational poverty and detriments to health (Brunt, 2013; Goosby and Heidbrink, 2013). These challenges will exist for the individual regardless of whether a casting agent knows that the actor is Black. However, an aspiring actor whom a casting agent infers to be Black may also face implicit bias or discrimination because of that perceived identity. Similar gatekeeping mechanisms may be activated in any of our creative domains when power brokers perceive an individual as having certain identities. In short, inferred identity plays a role in exclusion.

We restrict our study to gender and race/ethnicity. Other axes of demographic diversity include sexual orientation, gender presentation, disability, neurodiversity, age, religion, socioeconomic status, and more. We considered the possibility of studying LGBTQ+ identity and physical disability status but ultimately chose not to because of concerns about limitations of our approach, discussed in more detail below. As we will explain, our methodology hinges on having MTurk workers infer identity characteristics from publicly available information. However, individuals with certain identities may have compelling reasons not to make them public even beyond an inherent right to privacy. For instance, LGBTQ+ and disabled people may have concerns about discrimination, hate, and other social and professional consequences. In any case, our methodology would not infer someone to be LGBTQ+ unless they were publicly out. Similarly, our methodology would not infer someone to be physically disabled unless they chose to actively disclose a disability, or had limitations of mobility or functionality detectable to casual observers.

Even limited to axes of gender and race/ethnicity, demographic inferences are complicated. Gender is a construct that lives at the confluence of sociology, psychology, biology, and other factors (Kessler and McKenna, 1985; Ruble et al., 2007) and is still an active area of research. Racial categories lack a biological basis (Yudell et al., 2016), and furthermore, scholars still debate the meanings of race and ethnicity (Kincaid, 2018; Tibayrenc and Ayala, 2016). Despite the challenges of defining gender, race, and ethnicity, these concepts have tangible impacts, especially in the ways that they have been used as tools of oppression when ascribed to individuals and groups (Bederman, 2008; Collins and Bilge, 2016; Crenshaw, 1990; Kendi, 2016).

### Scope of study

We are interested in contemporary creative contributions. There are many domains one could consider including dance, literature, instrumental music, and more. As a convenience sample, we select four domains that have broad reach and available data: contemporary art, high fashion, box office film, and popular music. There are many potential loci of inquiry within these four creative domains. For instance, with regards to contemporary art, one might study diversity of museum curators, museum visitors, artists in commercial galleries, and more. In high fashion, one might study models, photographers, design house executives, and so forth. As we will now describe, our own interest is the individuals most directly responsible for artistic creation at high levels.

For contemporary museum art, we use data gathered in a previous study. Specifically, (Topaz et al., 2019) studied the socially inferred demographics of artists whose works were held in the collections of 18 major U.S. art museums at the time of data collection in 2017 and 2018. These museums are:

- Dallas Museum of Art,
- Denver Art Museum,
- Detroit Institute of Arts,
- High Museum of Art, Atlanta,
- Los Angeles County Museum of Art,
- Metropolitan Museum of Art, New York,
- Museum of Art, Rhode Island School of Design,
- Museum of Contemporary Art, Los Angeles,
- Museum of Fine Arts, Boston,
- Museum of Fine Arts, Houston,
- Museum of Modern Art, New York,
- National Gallery of Art, Washington D.C.,
- Nelson-Atkins Museum of Art, Kansas City,
- Philadelphia Museum of Art,
- San Francisco Museum of Modern Art,
- Whitney Museum of American Art, New York, and
- Yale University Art Gallery.

The original study retrieved 186,657 artist records from these museums’ websites and sampled 11,522 of them for investigation. Of those, 10,108 were found to correspond to individual identifiable artists; the remaining records were companies (*e.g.*, Tiffany Studios), collectives, collaborations, or unidentified artists. The data set also contains each artist’s decade of birth. Because of our interest in contemporary art, we restrict attention to artists born 1940 or later, of whom there are 2229 in the data set.

Within the fashion domain, we study high fashion designers showing on runways at the “Big Four” fashion weeks: London, Milan, New York, and Paris. Since three of these fashion weeks occur in Europe, one might ask whether they should be taken as proxies for high fashion within the United States. These fashion shows are the ones cataloged online in the Vogue Runway Archive (Vogue, 2015). While there exist several popular fashion and beauty outlets—including Cosmopolitan, Glamour, Allure, and Harper’s Bazaar—Vogue has, arguably, the strongest focus on high fashion. Vogue was founded in the United States and its U.S. edition has the largest circulation of any of the Vogue publications. Therefore, we accept the set of fashion designers covered in its Runway Archive as a proxy of influential high fashion in America.

A fashion house may show one or more of its lines (*e.g.*, couture, ready-to-wear) at one or more of the four fashion weeks during one or more seasons each year. Each show may have one or more primary designers. We study designers from the following 11 seasons and lines, across the four Fashion Weeks:

- Couture, Spring 2018, Fall 2018, Spring 2019, Fall 2019,
- Ready-to-Wear, Spring 2019, Fall 2019,
- Menswear, Fall 2018, Spring 2019, Fall 2019,
- Resort 2019, and
- Pre-Fall 2019.

The data set is constructed from 2608 fashion shows. On the Vogue Runway Archive website, each show has its own page with textual commentary and photos. We ask crowd workers to visit these pages, read the commentary, and extract the names of the designers. There are several cases to consider and we guide crowd workers on how to address these via examples:

- Example 1. The fashion house called Elie Saab had a fashion show. Based on your reading of the article, you discover that the designer is, in fact, Elie Saab. That is to say, the fashion house and the designer have the same name. So, you enter the designer’s name as Elie Saab.
- Example 2. The fashion house called Christian Dior had a fashion show. Based on your reading of the article, you discover that the clothing was designed by a person named Maria Grazia Chiuri who is discussed at length in the text provided. Therefore, you enter the designer’s name as Maria Grazia Chiuri.
- Example 3. The fashion house called Jean-Paul Gaultier had a fashion show. The linked article discusses at length someone named Stephen Jones, but reading the text and doing other research reveals that while Jones had some creative influence, that Jean-Paul Gaultier was the primary designer. Therefore, you enter the designer’s name as Jean-Paul Gaultier.
- Example 4. The fashion house called Sachin & Babi had a fashion show. The linked article and your research reveal that the designers are, in fact, Sachin Ahluwalia and Babi Ahluwalia, and that it is a collaborative design effort with both individuals making fairly equal contributions. Therefore, you enter the names of both designers.

Of course, many designers have work appearing in more than one show. After we remove redundant records, there are 889 unique fashion designers identified by the crowd workers.

For popular music, we use online data from Billboard, a major American media brand (Billboard, 1997). The Billboard Hot 100 songs are ranked each year based on sales, radio play, and streaming. The Billboard Top 200 Albums incorporate sales and streaming. The Top 100 Musical Artists are ranked based on a combination of album sales, song play, social media followers, and website views. We retrieve the musicians appearing on these lists for the 2018 and 2019 year-end charts. The listing could be for an individual’s name, a band’s name, or in certain cases such as a soundtrack album, there may be no single band or individual credited. We want to determine primary musical artists. As we did with fashion, we provide several examples to crowd workers to guide their identification of musical artists:

- Example 1: If the musical artist listed is Taylor Swift then the primary musician would be Taylor Swift.
- Example 2: If the musical artist listed is Maroon 5 then the primary musician would be the lead artist, Adam Levine.
- Example 3: If the musical artist listed is Cardi B, Bad Bunny & J Balvin for the song I Like It, then the primary musician would be Cardi B because the song appeared on her album.

The crowd workers identify 221 unique popular musical artists.

Within the film domain, we gather information from the Internet Movie Database (IMDB) (IMDB.com, 1990), and in particular, the 100 highest box office grossing films of 2018 and 2019. Films are created through the collaborative effort of many creative contributors ranging from set designers to actors. For each film, IMDB identifies ten principal cast and/or crew, and we take these principals to represent the most significant contributors. Amongst the 200 films that serve as the basis of our film data set, there are 1608 unique name-principal role pairs, broken down as 630 actors, 400 writers, 255 producers, 196 directors, 67 composers, 41 cinematographers, 13 editors, and 6 production designers. However, there are 26 individuals who have more than one principal role. For example, Rashida Jones appears in our data set as an actor for The Grinch and as a writer for Toy Story 4. As we are not interested in studying principal role, we eliminate duplicate names to obtain 1580 unique principals.

As we have described above, for fashion, contemporary art, and popular music, crowd workers enter the names of creative contributors as free text. These free text responses obey different conventions with regards to spacing, capitalization, punctuation, and spelling. To standardize names, we use the data tool OpenRefine to cluster and clean the text using several approaches including fingerprint merging, n-gram merging, and nearest-neighbor merging with Levenstein distance; see (Verborgh and DeWilde, 2013) for an introduction to these approaches. We arrive at a set of 2229 contemporary artists, 889 fashion designers, 1580 film cast/crew, and 221 popular musicians that serve as our focus for study. These 4921 records comprise 4895 individuals, as 24 individuals have creative contributions in two creative domains, yielding 48 unique name-domain pairs. We retain all of these pairs because we are interested in studying socially inferred demographics within each domain.

### Crowdsourced demographic inference

For contemporary artists, we use data collected in (Topaz et al., 2019). For fashion, film, and music, we collect new data. For each creative contributor admitted to our study as described above, we﻿ deploy to MTurk the individual’s name and a brief description of their creative contribution. We ask crowd workers to make demographic inferences about each individual based on Wikipedia searches, Google searches, personal information, or any other sources. Our two primary questions are:

1. What do you believe is the person’s gender? In answering this question, you might use the persons’s name, and/or any pictures you find of the person, and/or any textual information you find referring to the person with gendered pronouns (*e.g.*, they/he/she, theirs/his/hers).
  Woman
  Man
  Binary man/woman choice does not apply (e.g., nonbinary, gender nonconforming, agender)
  Cannot determine; I do not have enough information to have an informed belief
2. An ethnic group is a category of people who identify with each other based on similarities such as common ancestral, linguistic, social, cultural or national experiences. What do you believe is the person’s primary ethnic background?
  American Indian or Alaska Native. A person having origins in any of the original peoples of North and South America (including Central America), and who maintains tribal affiliation or community attachment.
  Black or African American. A person having origins in any of the black racial groups of Africa.
  Asian. A person having origins in any of the original peoples of the Far East, Southeast Asia, or the Indian subcontinent including, for example, Cambodia, China, India, Japan, Korea, Malaysia, Pakistan, the Philippine Islands, Thailand, and Vietnam.
  Latinx. A person of Cuban, Mexican, Puerto Rican, South or Central American origin, or similar.
  Native Hawaiian or Other Pacific Islander. A person having origins in any of the original peoples of Hawaii, Guam, Samoa, or other Pacific Islands.
  Middle East or North Africa. A person having origins in locations such as Algeria, Morocco, Egypt, Lebanon, or Syria.
  White. A person having origins in Europe.
  An ethnicity not listed above.
  Cannot determine; I do not have enough information to have an informed belief.

The definitions of race and ethnicity are not universally agreed upon, and their distinctions are not generally the subject of consideration by laypeople who serve as MTurk workers. For simplicity, in our survey instrument above, we use the terminology of ethnicity, keeping in mind that we are actually addressing joint racial/ethnic identities. Another limitation of our approach is that we do not address multiracial/multi-ethnic identities.

Each time a crowd worker makes an inference for a characteristic, we ask for their confidence in the belief they expressed, which they may indicate as “not very confident,” “somewhat confident,” or “highly confident.” As mentioned earlier, for robustness of results, four crowd workers independently research each creative contributor. We combine the multiple inferences into a final crowd inference as follows. We assign points to the confidence ratings, ranging from one point for “not very confident” to three points for “highly confident.” Therefore, for each demographic characteristic, there are up to 12 confidence points possible. We set a threshold of six points assigned to an inference in order to accept it into our data set. For example, if two out of four crowd workers infer an individual to be agender, gender nonconforming, or nonbinary (AGNN) with high confidence, one crowd worker infers the individual to be a man with medium confidence, and one crowd worker infers the individual to be a woman with medium confidence, we accept the crowd’s inference as AGNN. In cases where no demographic choice meets the six point threshold, we assign NA to represent missing data. Similarly, in cases where two choices each earn six points, we interpret the crowd as not being able to reach consensus, and we assign NA. For a small amount of data returned as NA by this process, we have members of our research team play the role of crowd workers, attempting to infer demographics. These team members are barred from analysis of the data.

During analysis of results, we will subsume responses of “Middle East or North Africa” into the category “An ethnicity not listed above” in order to make our data commensurable with American Community Survey data. Our final categories for socially inferred race/ethnicity are: American Indian/Alaska Native (AIAN), Asian, Black, Latinx, Native Hawaiian/Pacific Islander (NHPI), white, and Not Listed. We recognize the unfortunate limitations of the ACS racial/ethnic categories. These are broad categories that, ideally, would be broken down to represent individuals more faithfully. Merely as one example, a person of Japanese ethnicity and an person of Pakistani ethnicity would both be categorized as Asian, though those individuals may not feel ethnically proximate to each other.

After the inference process, 172 records (3.5%) are still missing a socially inferred gender and/or race/ethnicity. As we are studying socially inferred identities rather than true ones, and because we require both gender and race/ethnicity to study intersecting identities, we are forced to eliminate these from our study. We arrive at our final data set of 2092 contemporary contemporary artists, 866 fashion designers, 1569 film cast/crew, and 220 popular musicians. These 4747 records comprise 4724 individuals, as 23 individuals have creative contributions in two creative domains, yielding 46 unique name-domain pairs. We retain all these pairs because we are interested in studying socially inferred diversity within each domain.

### Data reliability

As mentioned throughout, we take several steps to ensure the quality of demographic inferences and we recapitulate those here. We allow our HITs only to be completed by experienced MTurk workers who are located in the United States (increasnig the likelihood that they are familiar with U.S. racial categories), who have high approval rates, and who have high counts of previously completed tasks. We provide the workers with examples and detailed instructions to guide them in their process. We have each creative contributor in our study researched by at least four independent workers, and we require workers to state a confidence rating for their response, allowing us to weight that response to arrive at an honest representation of the crowd’s consensus (or lack thereof). It is worth noting that methodologies similar or identical to this one have been used frequently in the published literature, including, for example, (Topaz et al., 2019).

Finally, we perform a data validation task. One of the authors of this study who was not involved in the initial stage of demographic inference subsampled 3% of creative contributors from each artistic domain, for a total of 142 contributors. The researcher inferred race and gender without accessing the crowdsourced inferences. In all cases, this author’s inferences agreed with the consensus inference of the crowd, suggesting robustness of results. We refer to this as robustness, rather than negligible error, because we are measuring inferred identities and not actual ones.

### Benchmark data

The American Community Survey (ACS) is a demographic survey conducted by the U.S. Census Bureau. To provide a point of comparison for our results, we use the ACS, recognizing the limitation that we are studying socially inferred demographics and the ACS reports self-identified ones. More specifically, we use the 2019 Public Use Microdata Sample for five-year estimates (U.S. Census Bureau, 2019).

For comparison with socially inferred gender, we use the ACS variable for sex. The closest comparison we are able to make is by equating male sex with the gender “man” and female sex with the gender “woman.” We recognize the harm done by this choice. The absence of self-identified gender data from ACS and especially the erasure of agender, transgender, gender nonconforming, and nonbinary individuals is unfortunate and should be rectified.

As for race/ethnicity, we begin with ACS variables for race and Hispanic origin and create our own derived variable with the following categories:

- American Indian/Alaska Native (AIAN) = American Indian or Alaska Native race regardless of Hispanic origin,
- Asian = Asian race regardless of Hispanic origin,
- Black = Black or African American race regardless of Hispanic origin,
- Latinx = White race with Hispanic origin not from Spain, a race not listed with Hispanic origin not from Spain, multiple racial identities with Hispanic origin not from Spain,
- Native Hawaiian/Pacific Islander (NHPI) = Native Hawaiian/Pacific Islander race,
- White = White race with no Hispanic origin or Hispanic origin from Spain,
- Not listed = Any combination of race and Hispanic origin not captured by the choices above.

Finally, we recognize that not all creative contributors in our four domains are U.S. based. One may ask if it is valid to compare demographics within these domains to those of the U.S. population at large. The art museums in our study are located in the U.S. While three of the four major fashion shows do not take place in the U.S., U.S. fashion houses play a significant role. Finally, Hollywood is located in the U.S., and the popular music charts are American music charts. In short, we recognize that the comparison of these artistic spheres to ACS data is not perfect, but we proceed while bearing the limitations in mind.

### Data ethics

The American Statistical Association (ASA) provides ethical guidelines for statistical practice (American Statistical Association, Committee on Professional Ethics, 2018), addressing over 50 issues related to professional accountability, data and methodological integrity, public transparency, responsibility to research subjects, and more. We have considered all guidelines in detail, and we now mention a few that play a special role in our study.

The ASA states that investigators should identify and mitigate any preferences “that might predetermine or influence the analyses/results.” With this directive in mind, we use survey questions developed in other peer-reviewed, published research (Topaz and Sen, 2016; Topaz et al., 2019, 2020) (noting that some of us are co-authors on those papers). The majority of our data comes from anonymous crowd workers. A small amount of data comes from research performed by members of our team, but there was a firewall between that research and analysis of the data.

Additionally, studies should use “selection or sampling methods and analytic approaches appropriate and valid for the specific question to be addressed, so that results extend beyond the sample to a population relevant to the objectives with minimal error under reasonable assumptions.” Our study is a convenience sample; we make no statistical inferences about the population. In other words, we identify certain creative spheres that have been studied in prior scholarship, and we choose proxies for contribution within these spheres on the basis of convenience. Rather than making any inferences about a population, we simply report summary statistics on our proxies.

The ASA rightfully notes that investigators must identify “the ultimate financial sponsor of the study, the stated purpose, and the intended use of the study results.” Our work was commissioned by Revolt Inc., a creative consulting agency in London. Their own work was commissioned by Lifewtr, a division of Pepsi. As presented to us, the purpose of the commissioned study was to shed light on marginalization of certain demographic groups in key creative spheres.

Finally, we note that because the creative contributors are public figures, because crowd workers use publicly available information for their research, and because the data they provide constitutes socially inferred demographics (and not demographic truth), our research is not human subjects research. While our research and public data do meet the relevant ethical standards, it is nonetheless important to us to take all steps possible to mitigate potential harm caused by the release of our data set. It is challenging to navigate the tension between, on one hand, mitigation of potential harm, and on the other hand, reproducibility. To mitigate harm, we have opted to redact the names of creative contributors from our publicly posted data set. To support reproducibility, we will provide the unredacted data to researchers who request it from the corresponding author.

## Results and discussion

Aggregate socially inferred demographics appear in Table 1. The first two columns specify socially inferred gender and race/ethnicity, the next four columns report our research results, and the last column provides the ACS estimates.

**Table 1:** Socially inferred gender and race/ethnicity in four creative domains: contemporary art, high fashion, box office film, and popular music, as obtained through the process described in section “Methodology.”

Socially inferred	Socially inferred	Art	Fashion	Film	Music	ACS
Gender	Ethnicity	(*N* = 2092)	(*N* = 866)	(*N* = 1569)	(*N* = 220)	
AGNN	AIAN	0.0%	0.0%	0.0%	0.0%	0.0%
	Asian	0.0%	0.0%	0.0%	0.0%	0.0%
	Black	0.0%	0.0%	0.0%	0.0%	0.0%
	Latinx	0.0%	0.0%	0.0%	0.0%	0.0%
	NHPI	0.0%	0.0%	0.0%	0.0%	0.0%
	Not Listed	0.0%	0.0%	0.0%	0.0%	0.0%
	White	0.0%	0.2%	0.0%	0.5%	0.0%
Man	AIAN	1.0%	0.0%	0.0%	0.0%	0.4%
	Asian	7.2%	6.8%	3.2%	1.4%	2.6%
	Black	3.0%	2.2%	6.0%	34.1%	6.1%
	Latinx	4.8%	1.7%	2.7%	4.5%	8.7%
	NHPI	0.0%	0.0%	0.2%	0.0%	0.1%
	Not Listed	1.1%	2.2%	0.8%	0.5%	1.4%
	White	54.5%	42.0%	60.7%	41.8%	30.0%
Woman	AIAN	0.7%	0.0%	0.0%	0.0%	0.4%
	Asian	2.2%	7.0%	1.0%	0.5%	2.9%
	Black	0.9%	0.5%	3.6%	5.9%	6.6%
	Latinx	1.0%	0.7%	1.3%	1.4%	8.5%
	NHPI	0.0%	0.0%	0.0%	0.0%	0.1%
	Not Listed	0.6%	1.0%	0.1%	0.0%	1.4%
	White	23.0%	35.6%	20.5%	9.5%	30.9%

The rightmost column provides estimates of the U.S. population as calculated from the American Community Survey (ACS). Note: *AGNN* agender, gender nonconforming, or nonbinary, *AIAN* American Indian/Alaska Native, *NHPI* Native Hawaiian/Pacific Islander, *Not Listed* an identity not captured by the other categories.

In interpreting our results, it imperative to keep in mind two qualifiers. First, over/under-representation and majoritization/minoritization are not synonymous. For instance, it is possible for a group to be overrepresented in a creative domain as compared to that group’s presence in the the U.S. population at large, but this does not mean that the group is majoritized, nor that it is free from experiences of marginalization and oppression. In fact, there is no creative domain in which members of any non-white ethnic group come close to outnumbering white people, and hence these groups are likely to experience the effects of a white power structure. Second, for brevity, we are omitting repeated use of the modifier “socially inferred” before gender and race/ethnicity for the remainder of this section. We caution the reader to remember that our study results are socially inferred, that demographics from the ACS are self-identified, and that we have made several assumptions and simplifications (discussed previously) in order to facilitate a comparison between the two.

**Fig. 1:**
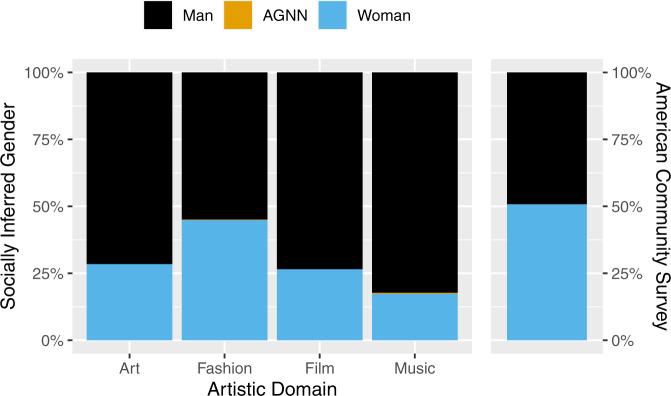
Socially inferred gender across four creative domains and sex data from the American Community Survey (ACS) (U.S. Census Bureau, 2019). As compared to the ACS, women are underrepresented in each domain. Note: AGNN agender, gender nonconforming, or nonbinary. See Table 1 for numerical data.

Women make up 51% of the U.S. population, and yet are underrepresented in each creative domain: contemporary art (28%), fashion (45%), box office film (27%), and popular music (17%). See Fig. 1, which displays gender in each creative domain alongside ACS data, and is convenient for comparing gender across domains and to the U.S. population. Fashion comes closest to meeting the U.S. population benchmark. Popular music is furthest, where women are underrepresented by a factor of three. Because of the necessity of using ACS binary sex data as a proxy for gender, we unfortunately cannot comment on the representation of genders other than man and woman.

**Fig. 2:**
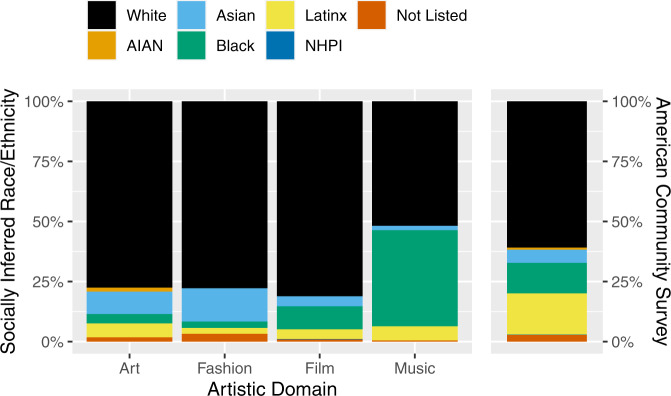
Socially inferred race/ethnicity across four creative domains and as derived from American Community Survey (ACS) data (U.S. Census Bureau, 2019). As compared to the ACS, marginalized racial/ethnic groups are underrepresented in most domains. Note: AIAN American Indian/Alaska Native, NHPI Native Hawaiian/Pacific Islander, Not Listed an identity not captured by the other choices. See Table 1 for numerical data.

As for race/ethnicity, non-white groups make up 39% of the U.S. population yet comprise approximately half that in contemporary art (22%), fashion (22%), film (19%); see Fig. 2, which displays socially inferred race/ethnicity alongside ACS data, and is convenient for comparing across domains and to the U.S. population. In contrast, marginalized ethnic groups represent 48% of creative contributors to popular music; the vast majority of that 48% are Black musicians, follwed by Latinx individuals. Latinx representation is low across all domains, but markedly lowest in fashion (2.4%), despite Latinx individuals comprising an estimated 17% of the U.S. population.

**Fig. 3:**
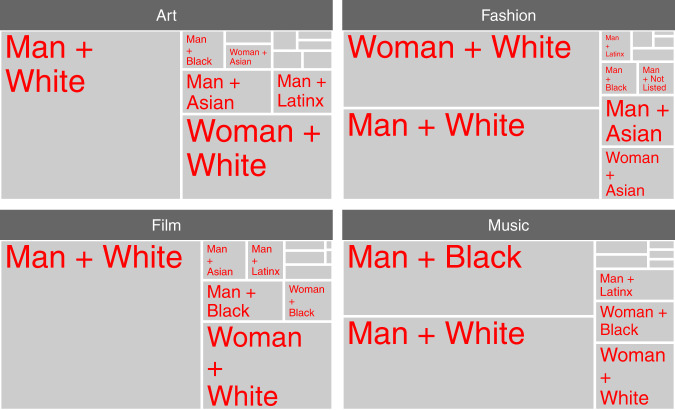
Socially inferred intersecting gender-ethnicity within each creative domain, where each light gray rectangle represents proportion within the domain. Art is dominated by individuals inferred to be white men, fashion by white men and women, film by white men, and music by black and white men. For readability, groups with small proportions are not labeled, but Table 1 provides the numerical data.

To provide a more detailed look at the intersection of gender and race/ethnicity within each domain, Fig. 3 displays proportional area charts. Each subdivision’s area represents the share of creative contributors belonging to an intersecting gender-racial/ethnic identity group, and the areas should be construed as summing to 100 percent. Each creative domain is dominated by one or two identity groups, and these always include white people and/or men. Contemporary art is dominated by white men (55%), followed by white women (23%). Fashion is dominated by white men (42%) and women (36%) at roughly similar levels. Film is dominated by white men (61%), followed by white women (21%). Popular music is dominated by men, with Black (34%) and white (42%) men comprising the largest shares.

**Fig. 4:**
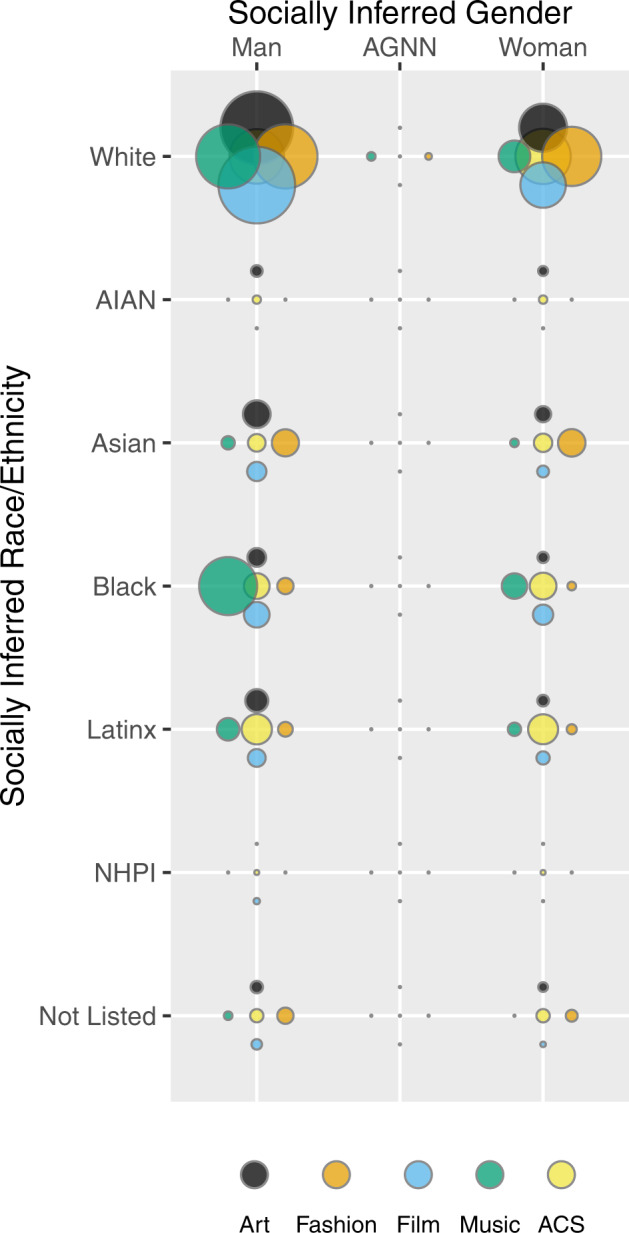
Socially inferred intersecting gender and ethnicity data compared across domains and to American Community Survey (ACS) data (U.S. Census Bureau, 2019). At each categorical intersection point, the black bubble represents the share of that identity group within the U.S. population, and the colored bubbles represent the shares within each creative domain. See Table 1 for numerical data. Note: AGNN agender, gender nonconforming, or nonbinary, AIAN American Indian/Alaska Native, NHPI Native Hawaiian/Pacific Islander, Not Listed an identity not captured by the other choices.

Finally, to compare gender-racial/ethnic identities across domains and to the U.S. population, Fig. 4 provides a bubble chart. At each categorical intersection point, the black bubble represents the share of that identity group within the U.S. population, and the colored bubbles represent the shares within each creative domain. White men are overrepresented in every domain as compared to the U.S. population. White women are slightly overrepresented in fashion and underrepresented in all other domains. Asian men are slightly overrepresented in art and fashion, and Asian women in fashion. Black men are substantially overrepresented in popular music. As stated previously, a group that is numerically overrepresented can still be minoritized and can still experience oppression. Beyond the aforementioned results, nearly all marginalized groups experience minoritization beyond the level in the U.S. population at large.

## Conclusion

With an eye towards the importance of diversity in creative fields both for popular audiences and for the creative contributors themselves, we have studied socially inferred gender and race/ethnicity in contemporary art, fashion, box office film, and popular music. As compared to American Community Survey data for the U.S. population, women are underrepresented in each creative domain. Marginalized racial/ethnic groups, taken in aggregate, are underrepresented by a factor of two across the board, with most underrepresented in several domains. As for the intersections of gender and race/ethnicity, white men are overrepresented in each domain, by a factor of 1.4 in fashion and music, 1.8 in contemporary art, and 2 in box office film.

Our study focuses on inferred demographic identities because self-identified data is unavailable and is unlikely to be obtained. Both ethically and scientifically, it would be preferable to have self-identified demographic data for the public figures in our study. Still, under-representation and marginalization in creative fields matter, and so we have proceeded—with caution—to study it empirically. Another limitation of our study pertains to our choice of data sources, namely, contemporary artists appearing in the catalogs of certain U.S. museums, designers of shows in major fashion weeks, principals on top-grossing box office films as tabulated on the Internet Movie Database, and popular musicians appearing on Billboard charts. We intend these sources to serve as reasonable proxies for creative contribution in each domain, but other choices of data are certainly possible, and may yield different results. Our study is also limited in the specific axes of identity addressed. The American Community Survey reports not gender, but rather, a binary sex, resulting in the erasure of many gender minority groups in their data and, by extension, our own study. Moreover, while our study addresses primary, socially inferred gender and race/ethnicity, it does not address multiracial/multi-ethnic identity, nor does it address many other important axes of identity.

Our study is empirical and speaks neither to the reasons for under-representation in creative domains, nor to the best solutions. However, our work *does* identify specific loci of under-representation, which might help prompt a closer look with solutions in mind. For instance, knowing that Latinx individuals are underrepresented by nearly a factor of three in film, or that Black women are underrepresented by a factor of 13 in fashion, can help scholars, activists, and professionals target their efforts and assess change. To change these numbers would require public pressure and/or the intervention of executives, influencers, and other power brokers.

## Data Availability

Though our research is not human subjects research, it is nonetheless important to us to take all steps possible to mitigate potential harm caused by the release of our data set, which contains social inferences of personal demographic characteristics. To mitigate potential harm, we have opted to redact the names of creative contributors from our publicly posted data set. This anonymized data is available in an institutional GitHub repository at https://github.com/qsideinstitute/creativecontributors. To support reproducibility, we will provide the unredacted data to researchers who request it from the corresponding author.
